# Localised Objective Characterisation Assessment of Lymphoedema (LOCAL): Using High-Frequency Ultrasound, Bioelectrical Impedance Spectroscopy and Volume to Evaluate Superficial Tissue Composition

**DOI:** 10.3390/diagnostics14151616

**Published:** 2024-07-26

**Authors:** Jennifer Sanderson, Neil Tuttle, Robyn Box, Hildegard Reul-Hirche, E-Liisa Laakso

**Affiliations:** 1 Menzies Health Institute Queensland, Griffith University, Gold Coast, QLD 4215, Australia; liisa.laakso@mater.uq.edu.au; 2 School of Health Sciences and Social Work, Griffith University, Brisbane, QLD 4111, Australia; n.tuttle@griffith.edu.au (N.T.); hildegard.reul-hirche@griffithuni.edu.au (H.R.-H.); 3 QLD Lymphoedema and Breast Oncology Physiotherapy, Brisbane, QLD 4051, Australia; 4 Royal Brisbane and Women’s Hospital, Herston, QLD 4006, Australia; 5 Mater Research Institute, University of Queensland, South Brisbane, QLD 4101, Australia

**Keywords:** lymphoedema, assessment, ultrasound, high-frequency ultrasound, bioimpedance, limb volume, tissue, characteristics, characterisation

## Abstract

Lymphoedema tissue is characterised by excess free fluid and structural changes to the extracellular matrix (ECM) in the form of fibrotic and fatty deposition. These tissue characteristics are integral to the assessment of lymphoedema progression; however, clinicians and researchers often focus on changes in the free fluid, volume and function of lymphatic vasculature to inform practice. Subsequently, little is known about the effect of clinical interventions on lymphoedema tissue composition. This article presents a novel approach to classify lymphoedema tissue. The Localised Objective Characterisation Assessment of Lymphoedema (LOCAL) classification combines diagnostic and clinically meaningful objective assessment thresholds to infer lymphoedema pathophysiological changes in tissue layers. The LOCAL classification method was verified using data from fifteen women with unilateral breast cancer-related lymphoedema who were evaluated at three sites on each arm using high-frequency ultrasound (HFUS), bio-electrical impedance spectroscopy (BIS) and volume measurements. Participants exhibited an uneven distribution of volume between the proximal and distal segments of the arm (*p* = 0.023), with multiple tissue compositional categories observed across sites on the same limb (*p* < 0.001). The LOCAL method demonstrated utility in categorising a diverse range of lymphoedema tissue layer changes beyond what can be ascertained from whole-limb measures.

## 1. Introduction

This article presents a novel approach to classify lymphoedema tissue which is utilised in an article by the same author group to evaluate the relationship between tissue composition and the pitting test [1].

Lymphoedema is a form of chronic oedema that develops due to impaired lymph uptake from the interstitium. As the lymphatic vasculature consistently fails to remove fluid from the tissue at an adequate rate, excess interstitial fluid volume accumulates, observed as localised or diffuse swelling [2]. A defining feature of lymphoedema is an increase in tissue layer volume. However, a measurement of limb volume change is indiscriminate of the unique structural changes that may occur within the tissue layers.

The complexity of lymphoedema assessment is associated with a cascade of cellular changes that occur in response to accumulated fluid. The lymphatic system has a role in transporting antigens, which are proteins associated with stimulating an immune response. Antigens are located on the surface of cells, such as viruses, bacteria and chemicals, and function as a signal to immune cells to initiate an immune response [3]. When fluid containing antigens accumulates in the interstitium rather than being transported through lymph vasculature, the immune system brings the immune response to the tissues. Consequently, there is an influx of inflammatory mediators, which are known factors in pathological changes developing within the lymphoedema tissue layer, including fibrosis and adiposis [4]. 

Lymphoedema can present with fluid accumulation, inflammation, fibrotic and adipose tissue compositional changes that may occur concurrently or predominantly alone [2]. Lymphoedematous tissue health deteriorates when the extracellular matrix (ECM) is replaced with fibrotic and adipose deposits. The compositional changes of the tissue layer develop into a physical barrier that is thought to impede tissue fluid exchange [1], contribute to cellulitis risk and recurrence, promote ongoing localised inflammation [5] and stimulate further pathological changes [5]. Lymphoedema tissue compositional change occurs in a remodelling process with various combinations of tissue characteristics able to occur at one time. With regards to treatment outcomes, tissues with predominantly excess free fluid are known to be most responsive to intervention, and tissues that comprise fibrotic and fatty deposition are less responsive to treatment [6]. However, there are limitations in the knowledge regarding the effect of clinical interventions on lymphoedema-induced tissue compositional changes, which is associated with the difficulties of tissue characterisation assessment. 

The characterisation of lymphoedema tissue is complicated by the considerable variation in tissue characteristics across patient populations and the pathological remodelling effect on the involved tissue. Effective clinical reasoning relies on an accurate assessment of tissue characteristics, but few objective assessment tools are readily available to clinicians that can characterise lymphoedema tissue layers and evaluate changes that may be occurring in those tissues at any one time.

The International Society of Lymphology (ISL) staging is recognized as the primary method for classifying lymphoedema [2]. However, confidence in the method is diminished by the subjective nature of the criteria; the use of the pitting test as a differentiating item when it is currently an unstandardised test; and the application of the grading system to the whole limb even when there is clinically evident variation in tissue characteristics between different sites in a limb [7].

Although assessment devices and tools are readily available to researchers and clinicians, objective measurements are yet to be incorporated into the ISL staging criteria [2]. Furthermore, few studies utilise objective measures to characterise the components of lymphoedema tissue to the degree that would confirm the ISL staging of research participants and could be translated into a clinical setting.

This study presents a new approach to classifying lymphoedema tissue using objective assessment items known to signify lymphoedema pathophysiological changes in tissue layers. To define criteria for each category, the method draws on previous research with respect to diagnostic thresholds and clinically meaningful values for high-frequency ultrasound (HFUS) echogenicity, bioelectrical impedance spectroscopy (BIS) R0 values indicating the extracellular fluid volume, and physical volume assessments. The method seeks to group lymphoedema sites with similar tissue characteristics using objective measurements.

## 2. Materials and Methods

Localised Objective Characterisation Assessment of Lymphoedema (LOCAL).

### 2.1. LOCAL Classification

The development of the LOCAL classification resulted from the necessity to group participants by similar lymphoedema tissue characteristics for a study by the same authors investigating the tissue features that enhance or resist a pitting response [1]. The foundation for LOCAL categories is derived from understanding the influence of lymphoedema pathophysiological progression on objective measurements and the clarity in clinical meaning that may be attained by using multiple assessment tools. 

Four categories of lymphoedema tissue characteristics were defined by objective criteria that support the presence of excess free fluid and the presence of tissue compositional changes (Table 1) [8].

‘No Oedema’ refers to the absence of objective signs of oedema. ‘Early Fluid’ refers to the presence of excess free-fluid as indicated by an increase in limb segment volume, an increase in segment BIS R0 ratio and confirmed with the hypo-echogenicity of the tissue layers in ultrasound imaging. Fibro-fatty change with a high fluid volume, i.e., ‘High Fluid+’, refers to signs of oedema presence with an increase in limb segment volume and an increase in the BIS R0 ratio and signs of tissue compositional changes indicated by the hyper-echogenicity of the dermal or subcutaneous tissue layers in ultrasound images. Fibro-fatty change with low fluid volume, i.e., ‘Low Fluid+’, is defined as limb segment volume and BIS segment R0 ratio measures within normal limits or disproportionate in magnitude or direction of change and hyper-echogenicity of the ultrasound images. 

The LOCAL categories are mutually exclusive and collectively exhaustive, whereby every lymphoedema site belongs to a single category as determined by meeting all criteria for the category. The criteria include objective measurement thresholds for the segment volume, segment BIS R0 ratio and ultrasound image echogenicity of the dermal and subcutaneous tissue layers:

- Segment volume parameters were based on the subclinical diagnostic thresholds utilised by previous authors. The inter-limb volume difference threshold is ≥5% for the dominant [9,10] and ≥3% for the non-dominant lymphoedema-affected limb segment. The latter threshold was considered sufficient for a subclinical change with reference to the non-dominant arm volume reported to average 3.3% smaller than the dominant limb [11]. Where baseline measures are available, a threshold of ≥3% limb segment volume change is used [2,12].
- The BIS R0 measure is most specific to the extracellular fluid volume component of the lymphoedema presentation [13]. R0 values for the LOCAL criteria were derived from diagnostic thresholds of the R0 ratio in segmental measures considering limb dominance [14,15]. Segment thresholds were calculated for the 20 cm segment size by averaging the 10 cm 2SD (standard deviation) R0 diagnostic thresholds for the correlating location. The R0 ratio thresholds in the LOCAL system are intended to distinguish between small and large differences in inter-limb extracellular fluid volume.
- The ultrasound echogenicity parameter is used to support the presence or absence of tissue compositional change. Lymphoedema tissue has been shown to become hypoechoic (darker) with diffuse fluid influx [15,16] and hyperechoic (lighter) with fibrotic and fatty deposition [17,18,19]. The distribution of hyper-echogenicity may encompass the full or partial cross-section of the tissue layer or appear as scattered flecks [17,18,19]. For this reason, a decrease in the echogenicity of the lymphoedema tissue layers was observed as fluid influx, and an increase in echogenicity escalated the classification to the fibro-fatty groupings.

The echogenicity outcome was chosen for simplicity of use in the LOCAL system as fibrotic and fatty deposition can occur concurrently in various proportions and ultrasound pattern recognition exceeds the scope of the LOCAL categorisation system. 

### 2.2. LOCAL Verification

#### 2.2.1. Patient Demographics

Data from fifteen women with breast cancer-related lymphoedema who participated in a dynamic ultrasound study investigating tissue responses to the pitting test were used for verifying the LOCAL categories [1]. People with unilateral lymphoedema were invited to participate. Exclusion criteria were pregnancy, a pacemaker, multiple limb lymphoedema, an incomplete first line of cancer treatment or being within three months of undergoing chemotherapy. Using the ISL consensus guideline, these participants were ISL stage I and II [2]. Demographic, lymphoedema and medical history were collected prior to the assessment (Table 2). 

#### 2.2.2. Ultrasound

High-frequency greyscale ultrasound assessments were performed using the Siemens Acuson S3000 (Siemens, Germany) ultrasound device with an 18 MHz linear transducer. Ultrasound gel was applied between the transducer, a single-use standoff (Aquaflex, 2 × 9 cm, Parker Labs, Fairfield, NJ, USA) and the skin.

Three sites were scanned on each participant’s lymphoedema-affected and contralateral limbs. Sites included the posterior forearm and anterior forearm with the participant seated, and the posteromedial upper arm was evaluated in the prone position. Location of assessment sites was standardised with respect to distance from the ulna styloid with forearm sites at 15 cm and the upper arm site at 30 cm. The lymphoedema site was assessed first and the image was optimised using the device gain (dB) and image-focusing tools. The contralateral comparative site for the same location was then assessed using the same device settings without further optimisation to maintain the comparability of measures.

Ultrasound images were analysed using the post-imaging processing software ImageJ version 1.50i (U.S. National Institutes of Health, Bethesda, MD, USA, https://imagej.nih.gov/ij/, 1997–2018, accessed 15 July 2016). Echogenicity was measured as the API from an operator-defined region of interest field within the tissue layer. Dermal and subcutaneous tissue layer echogenicity was recorded with tissue layers of comparable sites defined as hyperechoic (lighter) or hypoechoic (darker) if the affected tissue layer API was higher or lower, respectively. 

#### 2.2.3. BIS

BIS was measured using the Impedimed SFB7 device (ImpediMed, Brisbane, Australia), with current electrodes (Ag-AgCl, ImpediMed) positioned in locations for the whole arm as per manufacturer guidelines [20]. An additional electrode was applied to the medial aspect of the forearm 20 cm proximal to the ulna styloid to divide the limb into upper and lower segments. Impedance was measured in triplicate at 256 discrete current frequencies in the range of 3–1000 kHz. 

#### 2.2.4. Limb Segment Volume

Volume was calculated from circumferential tape measurements in 5 cm to 10 cm increments using a truncated cone formula. The measurement protocol followed the Australasian Lymphology Association guidelines [21]. Inter-limb volume differences were calculated for the 20 cm segment sizes.

#### 2.2.5. Distribution of Lymphoedema

The distribution of lymphoedema was evaluated by comparing inter-limb volume measures for the upper arm and forearm segments. Uneven volume distribution was defined as a >10% difference in the relative change between distal and proximal limb segments.

#### 2.2.6. Statistical Analysis

Statistical analyses were performed using the IBM SPSS version 25 (IBM Corp., Armonk, NY, USA). Descriptive statistics were used to report the demographic and categorisation results; Spearman’s correlation was used to test the relationship between the volume and BIS variables, and ISL and LOCAL classifications. The Wilcoxon Signed Rank Test was used to compare variables consisting of lymphoedema-affected and unaffected values.

Ethical approval was received from the Human Research Ethics Committees of Griffith University, Gold Coast (#2016/353) and the Centre for Advanced Imaging, The University of Queensland, Brisbane (#2016000887), where the research was performed. Informed and written consent was obtained from all participants. 

## 3. Results

A broad range of breast cancer presentations were evaluated as observed from participant demographics. With respect to factors that influence lymphoedema, the cohort included people with newly diagnosed to established lymphoedema, a range of cancer treatment regimens and participants with and without a history of cellulitis episodes. There was also a diverse representation of age and body mass index within the group which are factors known to influence tissue characteristics (Table 2).

Statistically, the lymphoedema-induced changes in tissue characteristics of the participant cohort comprised increased limb volume (*p* < 0.001) with an uneven distribution between proximal and distal segments of the arm (*p* = 0.023); and reduced echogenicity of the dermal layer (<0.001) (Table 3). 

Lymphoedema and contralateral limbs demonstrated a marked variation in tissue characteristics as indicated by high standard deviation values. When viewed as an uncategorised group, lymphoedema-induced tissue characteristics were inconsistent in the direction and magnitude of change compared to contralateral sites. The LOCAL method utilised these differences in the effect of lymphoedema on the tissue layers to subdivide the group by tissue characteristics (Table 4).

**Figure 1:**
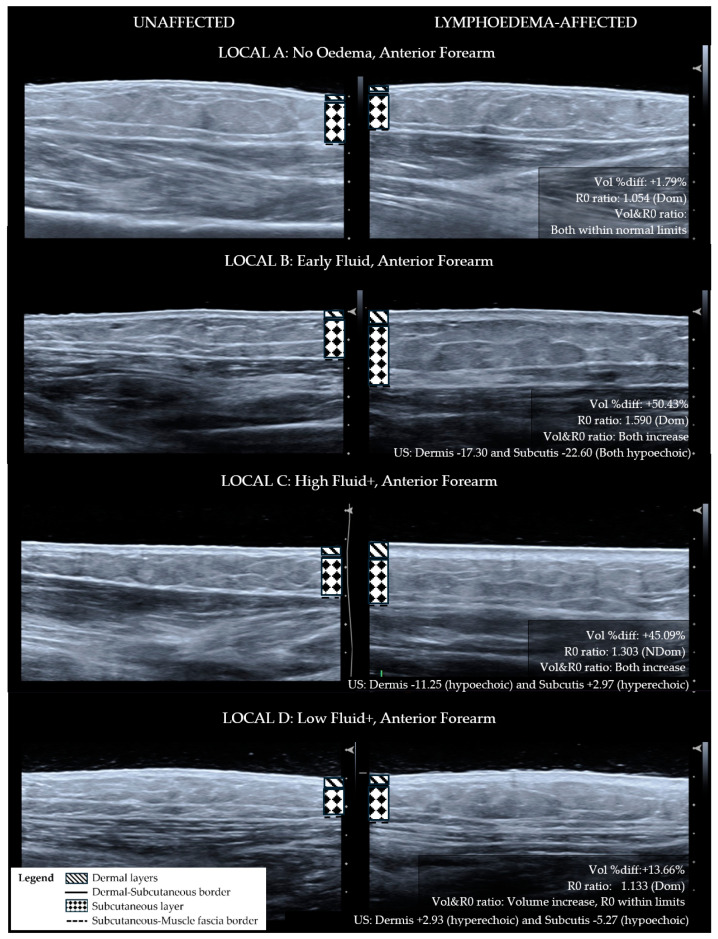
Ultrasound images for anterior forearm sites categorised with LOCAL criteria—matched lymphoedema-affected and unaffected sites. Lymphoedema-unaffected site (**left**), lymphoedema-affected test site (**right**). LOCAL A: No tissue characteristic change. LOCAL B: Lymphoedema dermal thickness increase and full-thickness hypo-echogenicity of the subcutis compared to the unaffected site. LOCAL C: Lymphoedema dermal thickness increase with hyperechoic change along the dermal–subcutaneous border and within the subcutis. LOCAL D: Lymphoedema dermal thickness increase with hyperechoic change along the dermal–subcutaneous border.

**Figure 2:**
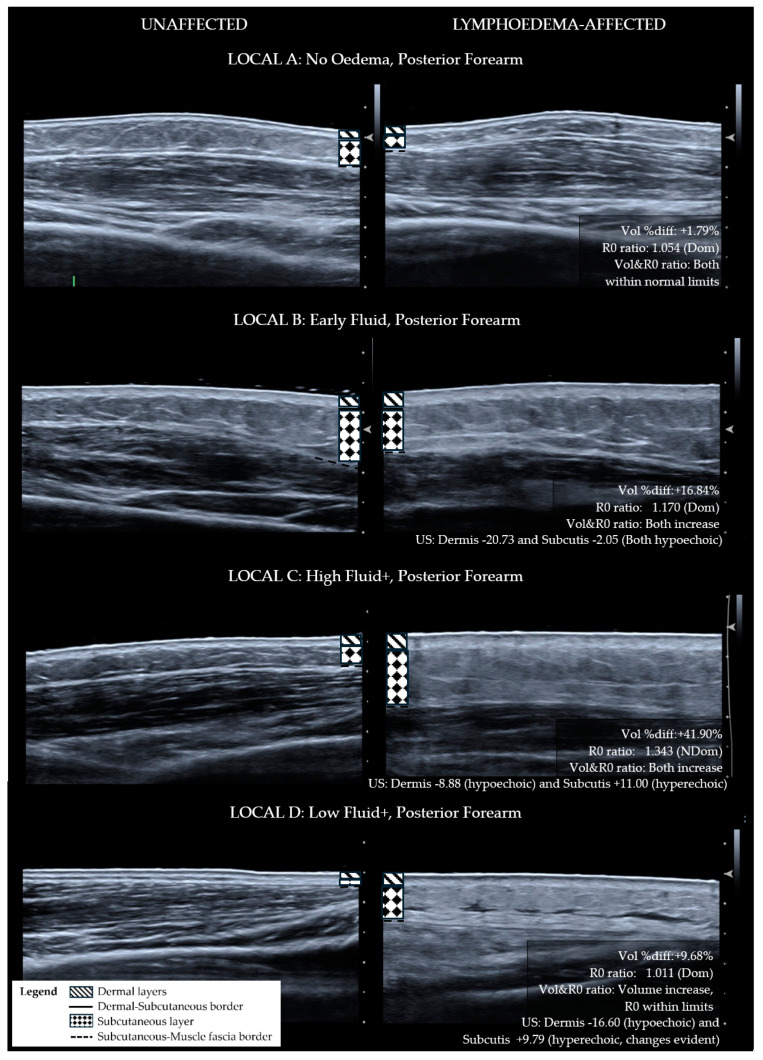
Ultrasound images for posterior forearm sites categorised with LOCAL criteria—matched lymphoedema-affected and unaffected sites. Lymphoedema-unaffected site (**left**), lymphoedema-affected test site (**right**). LOCAL A: No tissue characteristic change—the affected limb is also dominant which may explain lower subcutis and greater muscle thickness. LOCAL B: Lymphoedema dermal thickness increase and full-thickness hypo-echogenicity of the subcutis compared to the unaffected site. LOCAL C: Lymphoedema dermal thickness increase with hyperechoic change within the subcutis. LOCAL D: Lymphoedema dermal thickness increase with hyperechoic change along the dermal–subcutaneous border. Structural changes in fibrotic and fatty deposition have replaced normal tissue affecting the full thickness of the subcutis. Localised hypo-echogenicity within the subcutis is consistent with pools of fluid.

**Figure 3:**
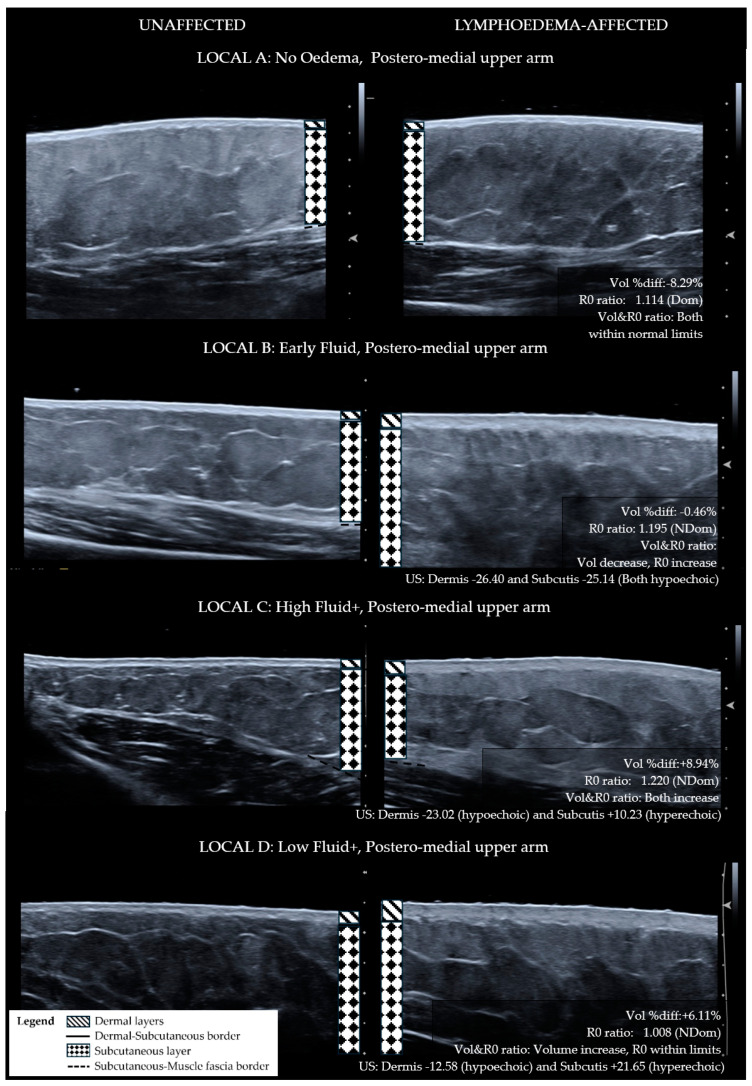
Ultrasound images for postero-medial upper arm sites categorised with LOCAL criteria—matched lymphoedema-affected and unaffected sites. Lymphoedema-unaffected site (**left**), lymphoedema-affected test site (**right**). LOCAL A: No tissue characteristic change. LOCAL B: Lymphoedema dermal thickness increase and full-thickness hypo-echogenicity of the subcutis compared to unaffected site. Serrated appearance of the dermal–subcutaneous border. LOCAL C: Lymphoedema dermal thickness increase with hypoechogenic changes to both tissue layers. Discord in echogenicity within the subcutis. LOCAL D: Lymphoedema dermal thickness increase with hyper-echoic serrated appearance of the dermal–subcutaneous border.

For the purpose of determining if ISL staging is sufficiently sensitive for diagnosing the lymphoedema tissue compositional change, ISL staging categories were compared with LOCAL groupings. To apply ISL staging, the limb is graded according to the worst affected area even if the whole limb was not affected. Using this method, the whole limb ISL stage did not correlate with the worst LOCAL category for the whole limb (r_s_ = 0.044, *p* = 0.877). The LOCAL criteria grouped each of the lymphoedema sites for each participant into broad categories pertaining to tissue layer characteristics as demonstrated in comparative ultrasound images (Figure 1—Anterior forearm, Figure 2—Posterior forearm, Figure 3—Postero-medial upper arm). By applying the LOCAL method, 57.8% of the evaluated sites were categorised into either LOCAL group C or D, indicating the presence of fibro-fatty tissue compositional changes (Table 1), which were not evident using the ISL staging criteria. Only one participant had the same LOCAL category evaluation for all three sites on their affected limb, and all other participants exhibited multiple LOCAL tissue categories between locations on the same limb.

## 4. Discussion

Lymphoedema is challenging to evaluate. Our aim was to understand lymphoedema tissue layer characteristics that may influence the clinical presentation and treatment outcomes utilising a novel approach to classify lymphoedema tissue more precisely in clinical practice. Other methods utilised for lymphoedema diagnosis and staging are considered subjective, cost prohibitive or unsuitable for clinical practice (Table 5).

The LOCAL method combines outcomes from objective measures to a group of research participants with similar tissue characteristics. The results verify previous research supporting the use of localised measures to inform on lymphoedema distribution [14,28] and clinical observations of variability in the lymphoedema effect between locations on the body. More importantly, the results suggest that the subjective clinical classification of lymphoedema can be error-prone without the benefit of instruments that can objectively define indicators of fibrotic and fatty tissue change. The authors suggest that it may be time to consider how objective criteria are used in combination with subjective observations to improve the accuracy of diagnosis and the grading of progression, evaluate the treatment effect and guide treatment options.

The characteristics of a lymphoedematous region change with the progression of the condition. Features that define the condition at one time point, such as excess free fluid in early lymphoedema, are inconclusive at a different time point, as occurs when fibrotic and adipose deposition replaces the ECM and excess free fluid is less evident [2]. Moreover, assessment of the effect of lymphoedema and its treatment within the tissue layers is hindered by substantial variation in tissue characteristics between individuals and sites in non-lymphoedema tissue. The authors contend that a greater understanding of tissue characteristics at different time points and different anatomical areas would aid in a client’s comprehension of the state of the condition and need for management and the clinician’s understanding of where and how to focus treatment.

The development of the LOCAL system derived from the authors’ concerns that the ISL staging criteria may be imprecise to group lymphoedema presentations by tissue layer characteristics. ISL classification does not account for the myriad of tissue changes that may occur in limbs affected by lymphoedema, whereas the LOCAL system provides a profile of tissue characteristics at different segments of the limb. The comparison of ISL staging with the LOCAL method indicates that the two tools evaluate different features of lymphoedema. Therefore, ISL staging and the LOCAL categories are not interchangeable; although, with further research, revision of the ISL staging criteria may eventually include objective items.

The LOCAL system is presented as preliminary work toward a clinically accessible method for grouping unilateral lymphoedema presentations objectively. The diversity of non-lymphoedema tissue in this study tested the ability of the LOCAL to differentiate lymphoedema-induced changes from individual tissue characteristics. Age [29,30], weight [31], medical history [32], cancer treatment and lymphoedema progression [18] are among many factors that relate to an individual’s tissue layer structure and the resultant effect of lymphoedema. The LOCAL method accounts for these known but difficult-to-quantify complexities of lymphoedema assessment by using comparative reference sites and setting thresholds based on expected indicators of progression. 

The LOCAL method was used successfully in a small dataset to categorise a broad range of lymphoedema tissue presentations differentiating between low and high extracellular fluid volume increases and the presence or absence of fibrotic and fatty tissue compositional changes. Ultrasound images illustrated inter-group differences and intra-group similarities, although the diversity of lymphoedema effect within LOCAL groups C and D was considerable, suggesting more categories or assessment items are required to further define the tissue characteristics.

Statistical analyses of lymphoedema datasets are susceptible to ambiguity when tissue characteristics are unaccounted for. Assessments used to evaluate the condition do not change linearly with disease progression, which can interfere with measures of the treatment effect. For example, in early-stage lymphoedema, an increase in ultrasound echogenicity or BIS R0 ratio values indicates increasing severity, and in later stages, a decrease in the same measurements indicates disease progression, which could be misinterpreted as an improvement in severity. The treatment effect will differ depending on the pathological tissue changes that are present; hence, the accurate classification of lymphoedema tissue is necessary for the appropriate interpretation of treatment outcomes.

In this study, ultrasound imaging demonstrated excellent potential for a detailed evaluation of lymphoedema. Lymphoedema tissue differentials of fluid influx, fibrotic deposition and fatty septa were able to be derived from the captured ultrasound images. However, the variation in tissue compositional changes complicated the use of these data in the LOCAL system. Many sites comprised multiple pathological tissue compositional changes concurrently, making the separation of excess fluid, fibrosis and adiposis tissue impractical. A separate tool or imaging technique would need to be developed to grade the tissue changes consistently and confirm the interpretation of high-quality ultrasound patterns. In recognition of the need for the future development of ultrasound protocols, the LOCAL system relies on the echogenicity measure for the simplicity of the tool and to maintain compatibility with the lower-quality imaging devices most accessible to therapists.

Lymphoedema can involve whole limbs and body regions, but whole-limb measures can dilute clinically meaningful information when the affected region is localised to a part of a limb. Localized assessment is supported by this research with high rates of uneven volume distribution between proximal and distal limb segments, multiple LOCAL categories observed on the individuals’ limbs and evident localized changes visualized in ultrasound imaging. This research is in agreement with previous authors concerning the utility of whole-limb versus segmental BIS (division of limb into sections), with the latter being more informative of the distribution of excess free fluid in the limb and more sensitive to small local changes [14,28]. In contrast to evidence supportive of segmental measures, whole limb measures are often used in monitoring programs for the early detection of lymphoedema, where the signs and symptoms are most likely to be obscure and localised [33,34]. The disparity in research findings and clinical methods is undoubtedly associated with the assessment time burden of localised measures compared to whole-limb measures. However, the momentary time saving may come at the expense of client outcomes, including earlier diagnosis, the detection of small regional changes or the identification of tissue compositional progression. 

### 4.1. Limitations

Although a diverse range of tissue compositions were captured in the dataset, the participant numbers were relatively small including mild to moderate severity lymphoedema presentations. This may limit the broader applicability of the research to lymphoedema of extreme severity. There is potential for a blinding bias as the investigators were unable to be blinded from lymphoedema-affected and -unaffected limbs.

### 4.2. Further Research

Refinement of the LOCAL criteria and the inclusion of more assessment items are expected to be required for the clinical use of this assessment tool. Further research is required to define the BIS and volume relationship with thresholds identifying when the two variables are not directly associated, thereby indicating the involvement of pathological changes in fibrosis or adiposis. We recognise that BIS is intended to assess early-stage lymphoedema; however, many clinicians continue to use the assessment in later stage lymphoedema to indicate a change in presentation with respect to the presence of extra-cellular fluid and to guide intervention.

## 5. Conclusions

The objective characterisation of tissue layers provides clinically meaningful information regarding lymphoedema. By understanding the pathophysiological state of lymphoedema tissue, we can better interpret assessment values and evaluate treatment efficacy. The LOCAL method provides a foundation for identifying lymphoedema-induced tissue compositional changes. The method was verified as capable of categorising a diverse yet small group of lymphoedema presentations using localised and segmented measures beyond what can be ascertained from whole-limb measures. Further refinement of the LOCAL method is warranted.

## Figures and Tables

**Table 1:** Localised Objective Characterisation Assessment of Lymphoedema (LOCAL) classification.

A No Oedema	B Early Fluid	C High Fluid+	D Low Fluid+
Site does not exhibit lymphoedema characteristics	Tissue exhibits extracellular fluid increase without compositional changes	Tissue exhibits fibro-fatty changes with high fluid volume	Tissue exhibits fibro-fatty changes with low fluid volume
Volume- Inter-limb segment difference (% difference between lymphoedema to non-lymphoedema segment) OR Limb segment change (% change from baseline)			
Within normal limits Inter-limb segment: <5.0% (Dom) <3.0% (NDom) OR Limb segment change: <3.0%	May or may not meet inter-limb threshold. OR Exceeds threshold for limb segment change: ≥3.0%	Greater than normal limits Inter-limb segment: ≥5.0% (Dom) ≥3.0% (NDom) OR Limb segment change: ≥3.0%	Greater than normal limits Inter-limb segment: ≥5.0% (Dom) ≥3.0% (NDom) OR Limb segment change: ≥3.0%
BIS R0- Inter-limb segment ratio (Unaffected/Affected)			
Within normal limits Forearm: ≤1.1385 (Dom) ≤1.0700 (NDom) Upper Arm: ≤1.1335 (Dom) ≤1.0915 (NDom)	Greater than normal limits Forearm: >1.1385 (Dom) >1.0700 (NDom) Upper Arm: >1.1335 (Dom) >1.0915 (NDom)	Greater than normal limits Forearm: >1.1385 (Dom) >1.0700 (NDom) Upper Arm: >1.1335 (Dom) >1.0915 (NDom)	Within normal limits Forearm: ≤1.1385 (Dom) ≤1.0700 (NDom) Upper Arm: ≤1.1335 (Dom) ≤1.0915 (NDom)
Volume and R0 relationship			
Both within normal limits	Both exceed thresholds OR R0 ratio increase and volume decrease, e.g., muscle atrophy or musculoskeletal cause	Both exceed thresholds	Both exceed thresholds. Disproportionate change, i.e., physical volume change large and R0 ratio relatively small OR Opposing direction of change, e.g., volume increase AND R0 ratio less than 1.000
Ultrasound Echogenicity—Lymphoedema API compared to Non-lymphoedema API			
N/A	Dermis: Hypoechoic AND Subcutis: Hypoechoic	Dermis: Hyperechoic OR Subcutis: Hyperechoic	Dermis: Hyperechoic OR Subcutis: Hyperechoic

Note. Dom = the dominant limb is also lymphoedema-affected. Ndom = the non-dominant limb is lymphoedema-affected. API = average pixel intensity. N/A = echogenicity results are not applicable for this category.

**Table 2:** Participant demographics.

Demographics		Range (Mean)
Age (years)		39–70 (58.8)
BMI (kg/m^2^)		19.25–43.98 (27.4)
Duration since Lymphoedema Onset (years)		0.25–10 (4.0)
Arm Dominance		n (%)
	Ipsilateral to lymphoedema	10 (66.7)
	Contralateral to lymphoedema	5 (33.3)
Surgical Procedure	Local breast excision + AD	5 (33.3)
	Mastectomy + AD	9 (60.0)
	Double mastectomy + unilateral AD	1 (6.7)
Adjuvant Treatment	Radiation only	2 (13.3)
	Chemotherapy only	2 (13.3)
	Both	10 (66.7)
	Neither	1 (6.7)
Episodes of Cellulitis	Never	11 (73.3)
	Once	1 (6.7)
	Multiple episodes	3 (20.0)
ISL Stage	1	6 (13.3)
	2	2 (40.0)
	2—late	7 (46.7)
	3	0

Note. AD = axillary dissection.

**Table 3:** Tissue characteristics.

Tissue Characteristic		Mean (SD)		*p*-Value ^#^
Volume	% diff inter-limb segment	15.74 (16.08)		-
Volume distribution	Uneven ≥ 10%	53.30%		0.023 *
	Even < 10%	46.70%		
Volume (mL)		Lymphoedema- affected	Unaffected	
	Whole limb	2825.49 (474.84)	2493.96 (423.96)	<0.001 *
	Distal segment	887.36 (197.76)	746.30 (135.81)	<0.001 *
	Proximal segment	1932.79 (337.17)	1753.01 (337.64)	0.012 *
Ultrasound echogenicity	Dermal	174.87 (24.67)	188.30 (18.27)	<0.001 *
	Subcutaneous	115.64 (32.67)	118.70 (34.42)	0.119
BIS	R zero whole limb	292.61 (61.95)	334.94 (43.09)	0.003 *
	R zero segment			
	Distal	136.42 (33.93)	160.02 (28.54)	0.004 *
	Proximal	176.82 (37.97)	187.89 (25.45)	0.109
	R zero ratio	1.161 (0.209)		-

^#^ Wilcoxon Signed Rank Test; * Statistically significant to a 0.05 level.

**Table 4:** Categorisation of arm lymphoedema participants using the LOCAL criteria.

LOCAL Category	Anterior Forearm *n* (%)	Posterior Forearm *n* (%)	Upper Arm *n* (%)
A. No Oedema	1 (6.6)	1 (6.6)	4 (26.7)
B. Early Fluid	6 (40.0)	4 (26.7)	3 (20.0)
C. High Fluid+	4 (26.7)	6 (40.0)	4 (26.7)
D. Low Fluid+	4 (26.7)	4 (26.7)	4 (26.7)
Total (n)	15 (100)	15 (100)	15 (100)

**Table 5:** Lymphoedema staging tools.

Description	Staging Details
ISL staging [2]	
Staging criteria include pitting, effect of limb elevation on oedema and skin changes. Easy to apply in clinical practice. Refers to tissue compositional changes but does not include objective criteria to confirm classification.	Stage 0—Subclinical, asymptomatic with known lymphatic transport impairment. Subtle symptoms and tissue changes may occur. Stage I—Early accumulation of fluid that improves or temporarily resolves with limb elevation. Pitting may occur. Stage II—Structural changes occurring within tissue layer. Oedema does not subside with elevation alone. Pitting evident up until late Stage II when excess subcutaneous fat and fibrosis develop. Stage III—Further fibrotic and fatty deposition, encompasses lymphostatic elephantiasis. Trophic skin changes. May or may not pit [2].
Lymphoscintigraphy	
Involves injection of a radiotracer into the interstitial space of the hand or foot. Images are taken over a period to indicate migration of the tracer from the tissue space through lymphatic vasculature and nodes [22]. Assesses lymphatic fluid transport through vessels and uptake to lymphatic nodes. Assessment is lymphatic-specific for lymphoedema differential diagnosis. Can evaluate lymph transit time, dermal backflow, identify asymmetrical node uptake and collateral vessels. Can identify sentinel nodes for surgery [23]. Does not evaluate tissue layer composition changes.	Multiple protocols published with various staging criteria [22] (p. 42). The Taiwan Lymphoscintigraphy Staging tool for unilateral extremity lymphoedema classifies the imaging results into patterns of lymph node uptake at proximal and intermediate sites on the limb, then subclassifies the drainage pattern into seven stages that are encompassed within three categories: normal drainage (L-0), partial obstruction (P1, P2, P3) and total obstruction (T4, T5, T6) [24].
Indocyanine green lymphangiography (ICG-L)	
Indocyanine green is injected intradermally and the fluorescence observed using near-infrared optical imaging. Assesses lymph uptake, transportation, drainage pathways and dermal backflow patterns. Used to inform surgical options [25]. Does not evaluate tissue composition changes.	MD Anderson ICG-L staging has 6 stages that are defined by observations of lymphatic patency and the pattern of dermal backflow. Stage 0: Normal linear lymphatics and no dermal backflow Stage 1: Many patent lymphatics and minimal lymphatic dermal backflow Stage 2: Moderate number of patent lymphatics and segmental dermal backflow Stage 3: Few patent lymphatics and extensive dermal backflow Stage 4: Dermal backflow involving the hand Stage 5: No proximal uptake of ICG from the injection site [25]. Dermal backflow patterns termed splash, stardust and diffuse, have been observed to correlate with lymphoedema severity [26].
Magnetic resonance lymphangiography	
Magnets and radio waves are used to produce detailed images of the body including soft tissue, muscle, bone and blood vessels. When a contrasting agent is used lymphatic vessel appearance and function can be evaluated [27]. High-quality imagery technique with sequence options to clearly differentiate tissue layer composition.	Multiple grading options are described to classify components of lymphoedema presentations including fluid, fat, appearance and function of lymph vessels, honeycombing, dermal thickness and oedema distribution. Leg lymphoedema staging published by Salehi et al., 2023): Fluid accumulation grade: 0 = no fluid 1 = honeycombing/reticular pattern of fluid within the subcutaneous fat 2 = continuous visible stripe of fluid between the fat and the muscle fascia Fat accumulation grade: 0 = no excess fat 1 = fat accumulation less than twice the width of the widest fat stripe on the unaffected side 2 = fat accumulation greater than twice the width of the widest fat stripe on the unaffected side [27]

## Data Availability

The data presented in this study are available on request from the corresponding author J.S.
